# Lack of FLT3-ITD in Tunisian childhood acute lymphoblastic leukemia

**DOI:** 10.4314/ahs.v22i2.35

**Published:** 2022-06

**Authors:** Rim Frikha, Nawel Abdellaoui, Olfa Kassar, Tarek Rebai

**Affiliations:** 1 Department of Medical Genetics-HediChaker Hospital, Sfax-Tunisia; 2 Laboratory of Histology, Faculty of Medicine of Sfax, Sfax-Tunisia; 3 Departments of Hematology, HediChaker Hospital, Sfax-Tunisia

**Keywords:** Fms-like tyrosine kinase 3, internal tandem duplication, acute lymphoblastic leukemia

## Abstract

**Background:**

The fms-like tyrosine kinase 3 (FLT3) gene belong to the class III receptor tyrosine kinases witch is predominantly expressed on hematopoietic progenitor cells, and plays an important role in haematopoiesis. Targeting the FMS-like tyrosine kinase receptor-3 (FLT3) in acute leukemia is mainly important. Therefore, activating mutations in FLT3, primarily the FLT3-internal tandem duplication (FLT3-ITD), was used as a prognostic marker especially in myeloid leukemia; however, in ALL, the prognostic relevance of FLT3 mutations is less clear.

**Objectives:**

This study was conducted to evaluate the frequency of FLT3-ITD mutation in Tunisian childhood acute lymphoblastic leukemia, and to correlate this mutation with prognostic parameters.

**Methods:**

Genomic DNA was extracted from EDTA-anticoagulant blood samples from a total of 25 children suffering from acute lymphoblastic leukemia (ALL). After DNA extraction, the polymerase chain reaction using specific primers was conducted to screen the FLT3-ITD.

**Results:**

In acute lymphoblastic leukemia (ALL), 9 cases with LAL-B were detected and the median age is 13 years. Chromosome abnormalities were detected in 5 with ALL and are correlated with worse prognosis (very high risk and relapse). At molecular lever, never FLT3-ITD was detected.

**Conclusions:**

Our findings suggest that FLT3 mutations are not common in Tunisian childhood ALL and thus do not affect clinical outcome.

## Introduction

The fms-like tyrosine kinase 3 (FLT3) gene belongs to the class III receptor tyrosine kinase which is predominantly expressed on hematopoietic progenitor cells, and plays an important role in hematopoiesis^1^, ^2^. Two distinct forms of FLT3 mutations have been identified, internal tandem duplication (ITD) in the juxta-membrane domain and a point mutation within the activation loop of the tyrosine kinase domain (TKD) which mostly affects aspartate 835 (D835). Both mutations are thought to be involved in leukemogenesis by constitutively activating the receptor 1, 2. Internal tandem duplication (ITD) in the fms-like tyrosine kinase 3 (FLT3) gene is the most common abnormalities in acute myeloid leukemia (AML) with a range of approximately 5–15% of children and 25–35% of adults^3^–^6^. A poor prognosis is associated with this mutation 7, 8.

In recent years, FLT3 has been a subject of several studies as prognostic marker mainly in AML patients. However, in ALL, The result is under debate 5, 9. Moreover, no data is available in Tunisian patients.

Therefore, we carried out this study to assess the prevalence and the prognostic significance of FLT3 ITD in Tunisian children with ALL.

## Materials and Methods

A retrospective study of review of ALL patients was performed. An informed consent was taken from all the persons participating in this study.

### Patient Samples

This study included 25 children with acute lymphoblastic leukemia who had attended the Departments of Hematology at the university hospital of Southern of Tunisia. The bone marrow samples of all patients were evaluated morphologically according to the French-American-British (FAB) classification. After morphological and immunological diagnosis was made, EORTC protocol was started. The ALL patients were divided into 3 groups based on the response to 2 mg/kg predniolone therapy on the 8^th^ day of treatment: standard risk group (SRG), medium risk group (MRG) and high-risk group (HRG). Correlations to other biologic factors such as karyotype and leukocyte count were also considered.

### Laboratory Methods

Genomic DNA was extracted from EDTA-anticoagulant blood samples according to the salting methods. After DNA extraction, the polymerase chain reaction using specific primers was conducted to screen the FLT3-ITD. The amplification performed on the Mini MJ (Bio-Rad) and entailed an initial denaturation of 94 oC for 5 minutes, followed by 35 cycles of denaturation at 94 oC for 30 seconds, annealing at 60 oC for 30 seconds, and extension at 72 °C for 1 minute, with a final extension at 72 °C for 5 minutes.

### Statistical analysis

All statistical analysis was done using SPSS ver. 17 (statistical package for social sciences) software.

## Results

The mean age of ALL patients was 13 years (range 5–18 years). Of ALL patients, 10 patients (40%) were boys, and 15 patients (60%) were girls. Of these patients, ten were in MRG, five patients were in SRG, and ten patients were in HRG group. During chemotherapy, 5 patients (20%) relapsed during follow up. Of the patients who relapsed, 2 patients died and 3 are still alive. Eight (32%) ALL patients were in induction or consolidation and the rest (n=12; 48%) were on maintenance treatment. The distribution of the patients according to FAB morphologic classification and properties of their final status are shown in Table 1.

**Table 1:** clinical characteristics of ALL patients

	N (number)	%
Male	10	40
Female	15	60
SRG	5	20
MRG	10	40
HRG	10	40
Subgroups (FAB)	L1 L2 L3 5 10 15	L1 L2 L3 20 40 40
Relapse	5	20
With induction/ consolidation treatment	8	32
With maintenance treatment	12	48
Dead	2	8

SRG: standard risk group; MRG: medium risk group; HRG: high risk group; FAB; French-American-British

**Figure 1:**
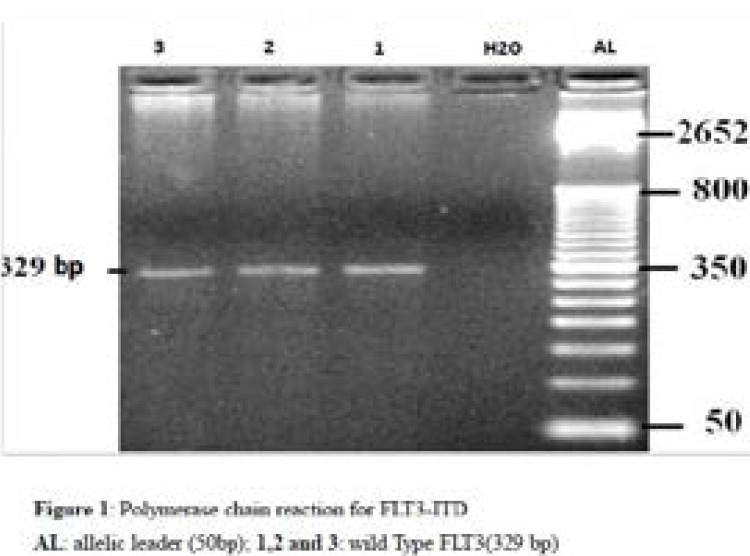


We checked for FLT3/ITD mutation among acute lymphoblastic leukemia children, but it was not found. (Figure 1)

## Discussion

Currently, this is the first data on FLT3 mutations in Tunisian children with acute lymphoblastic leukemia (ALL). No FLT3 ITD mutation was found. This result is in the range of reported frequency 5, 10, and ALL patients carry up to12.5 % and 4 % FLT3-ITD mutations; respectively for childhood and adulthood (Table 2).

**Table 2:** Review of literature among frequency and prognosis of FLT3 ITD both in pediatric and adults

Country	Number of cases	Mutation FLT3/ITD (%)	Comments and references
Saudi Arabia	77 cases 29 adult- 48 children	2% in children	In pediatric ALL: No prognosis significance 5
Turkey	80 children	7.5	In pediatric ALL: No prognosis significance 9
Iraq	25 cases: 9 adult -16 child	12,5% in children	In pediatric ALL 12
Iran	73 children	4.1	In pediatric ALL 13
Asia	381 children	0.2	In pediatric: Significance association with hyperdiploid 14
Pakistan	25 adults	4	In adult ALL No prognosis significance 15
China	83 adults 61 adults	0 3.2	In adult ALL: No prognosis significance 16–18

Nevertheless, it has been demonstrated that activating mutations of the FLT3 receptor tyrosine kinase are common in acute myelogenous leukemia (AML) and they are found in approximately 5–15% of children and 25–35% of adults with AML. Moreover, FLT3-ITD had negative impact on patients with AML 7, 8. However, targeting FLT3-ITD will not be beneficial in ALL and a lager number of Tunisian ALL patients are necessary to evaluate the prognostic effect of this mutation.

In interpreting our results, some limitations need to be addressed. First, the FLT3 assay was used as a qualitative diagnostic tool only in peripheral blood sample of ALL patients. To sensitively and accurately detect FLT3-ITD, correlation between peripheral blood and bone marrow regarding FLT3-ITD status should be investigated.

Secondly, the limited sample size of this study doesn't allow statistical correlation with biological parameters. In fact, occurrence of the FLT3-ITD has been strongly associated with higher peripheral leukocytes and higher blast percentages both in PB and BM 4, 11. It will be necessary to expand the sample size and consider biological factors for exploring this relationship. Finally, FLT3 is rarely mutated in leukemic lymphoblasts and the prognostic relevance of FLT3 mutations is less clear. Finally, despite the pathogenic effect of this mutation in hematopoietic proliferation and differentiation result, it cannot cause acute leukemia by itself and requires other genomic alterations related to cell differentiation.

Moreover, our study highlights that this molecular assay is an easy accurate and reliable test. Nevertheless, as long as there are no substantial studies reporting the importance of the mutation in ALL and its link with the prognosis it is not possible to establish a routine screening of FLT3-ITD in ALL patients.

## Conclusion

Our findings suggest that FLT3 mutations; mainly the FLT3-ITD; are not common in Tunisian childhood ALL and thus may do not be affecting clinical outcome.
